# Switching PD‐1 to BRAF + MEK inhibition improves recurrence‐free survival in patients receiving a second course of adjuvant melanoma therapy

**DOI:** 10.1111/jdv.20708

**Published:** 2025-05-07

**Authors:** Katharina Schumann, Kai Christian Klespe, Cornelia Mauch, Carmen Loquai, Ulrike Schultheis, Sevil Börger, Alexander Thiem, Steffen Emmert, Magdalena Hoellwerth, Peter Kölblinger, Van Anh Nguyen, Marina Wanner, Erika Richtig, Wiebke K. Peitsch, Wolfgang Harth, Veronika Zenderowski, Andreas Dominik Braun, Miriam Mengoni, Reinhard Dummer, Johanna Mangana, Lara Valeska Maul, Frank Meiß, Klemens Rappersberger, Oana Diana Persa, Tilo Biedermann, Christian Posch

**Affiliations:** ^1^ Department of Dermatology and Allergy, School of Medicine, German Cancer Consortium (DKTK) Technical University of Munich Munich Germany; ^2^ Department for Phlebology and Venerology Artemed Hospital Munich Germany; ^3^ Department of Dermatology and Allergology University Hospital RWTH Aachen Aachen Germany; ^4^ Department of Dermatology and Venereology University Hospital Cologne Cologne Germany; ^5^ Center for Integrated Oncology Aachen Bonn Cologne Düsseldorf (CIO ABCD) Cologne Germany; ^6^ Department for Dermatology and Allergology Clinic Bremen Ost Bremen Germany; ^7^ Department for Dermatology Johannes Gutenberg‐University Mainz Mainz Germany; ^8^ Department for Dermatology University Hospital Kassel Kassel Germany; ^9^ Clinic and Policlinic for Dermatology and Venereology University Medical Center Rostock Rostock Germany; ^10^ Department for Dermatology and Allergology Paracelsus Medical University Salzburg Austria; ^11^ Department for Dermatology, Venereology and Allergology Medical University Innsbruck Innsbruck Austria; ^12^ Department of Dermatology and Venereology Medical University Graz Graz Austria; ^13^ Department of Dermatology and Venereology Vivantes Skin Cancer Center Berlin Germany; ^14^ Department of Dermatology University Hospital Regensburg Regensburg Germany; ^15^ Department of Dermatology University Hospital Magdeburg Magdeburg Germany; ^16^ Dermatology Clinic University Hospital Zurich and University of Zurich Zurich Switzerland; ^17^ Department for Dermatology and Venereology Medical Center – University of Freiburg, Faculty of Medicine Freiburg Germany; ^18^ Department for Dermatology Clinic Landstraße Vienna Austria; ^19^ Faculty of Medicine Sigmund Freud University Vienna Vienna Austria; ^20^ Department for Dermatology, Clinic Hietzing Vienna Healthcare Group Vienna Austria

## Abstract

**Background:**

PD‐1 or BRAF + MEK inhibition is considered the current gold standard in adjuvant melanoma therapy. Little is known if, after the recurrence of the disease and surgery, a second course of adjuvant therapy might be beneficial.

**Methods:**

A multicenter, retrospective study investigating a second course of adjuvant therapy after recurrence and surgery in stage III–IV melanoma patients. Patients received nivolumab (NIV), pembrolizumab (PEM) or dabrafenib plus trametinib (D + T) between 01/2017 and 10/2021. The primary endpoint was 12‐month recurrence‐free survival (RFS2). Further analyses included descriptive and correlative statistics.

**Results:**

Sixty‐six patients from 22 centers in Germany, Austria and Switzerland were included. Thirty‐two patients received D + T as second‐course adjuvant therapy, 9 patients received PEM and 25 patients received NIV. Recurrence‐free survival for the second‐course adjuvant treatment (RFS2) was assessed after 12 and 24 months and showed a superiority of adjuvant BRAF + MEK over PD‐1 therapy (12‐months RFS2: 90.6% vs. 70.6%, HR 4.226 [95% CI 1.154–15.48]; *p* = 0.030; 24‐months RFS2 71.9% vs. 52.9%, HR 3.154 [95% CI 1.374–7.242]; *p* = 0.007). There was no significant decrease in OS with either BRAF + MEK or PD‐1 treatment (12‐months OS: 100% both, 24‐months OS: 100% vs. 93.8%).

Furthermore, therapy sequences were investigated. For better comparability, only BRAF V600 mutated patients were assessed: RFS2 was significantly better for patients with a class switch from PD‐1 to BRAF + MEK compared to BRAF + MEK to PD‐1 (HR 4.401 (1.04–18.63), *p* = 0.044). No new safety signals were detected.

**Conclusions:**

In the investigated cohort, a second course of adjuvant melanoma treatment is feasible and provides similar RFS compared to an initial course of adjuvant therapy using BRAF + MEK inhibitors; however, RFS2 is reduced for PD‐1 antibodies. In addition, both treatments were convincing with a 24‐month OS of almost 100%. Switching from adjuvant PD‐1 to BRAF + MEK treatment provided better overall RFS compared to switching from adjuvant BRAF + MEK to PD‐1 treatment.

## Graphical abstract

66 patients from 22 centers received adjuvant treatment after relapse under or after previous adjuvant therapy with BRAF + MEK or PD‐1. Data highlight a significant benefit when switching from PD‐1 to BRAF + MEK adjuvant therapy compared to a switch from BRAF + MEK to PD‐1 adjuvant regimens.

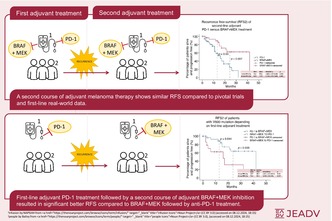

**Why was the study undertaken?**

**Lack of data:**

There are currently no data on second‐course adjuvant therapies for recurrence after previous adjuvant treatment of malignant melanoma.

**Clinical relevance:**

PD‐1 and BRAF + MEK inhibitors are the gold standard in adjuvant melanoma therapy. Optimized sequencing of adjuvant immune‐ and kinase‐targeted therapy has the potential to improve melanoma patient care.

**What does this study add?**

**Comparison of recurrence‐free and overall survival:**

This study shows similar RFS and OS outcomes of a second course of adjuvant melanoma treatment compared to the first course adjuvant treatment.

**Substance selection:**

The PD‐1 antibodies Nivolumab and Pembrolizumab and the kinase inhibitor combination Trametinib and Dabrafenib are approved for the adjuvant treatment of stage III melanoma.

**What impact does this study have on the understanding of disease and/or clinical care?**

The study provides first data that a class switch of PD‐1 to BRAF + MEK inhibition provides improved RFS compared to a switch from BRAF + MEK to anti PD‐1 treatment in an adjuvant setting.

## INTRODUCTION

Systemic melanoma therapy has undergone a revolution over the last several years. Starting with first improvements in survival treating stage IV disease using the CTLA‐4 inhibitor ipilimumab, immune‐oncological approaches with PD‐1 antibodies, as well as targeted approaches using kinase inhibitors have significantly improved recurrence‐free and overall survival.^1^, ^2^ Today, modern systemic melanoma treatment is also considered a gold standard in adjuvant settings: In metastatic, operable malignant melanoma stage II–IV, the tumour and/or metastases are completely resected and followed by systemic therapy.^3^, ^4^

Nivolumab and Pembrolizumab, both PD‐1 checkpoint inhibitors, as well as Dabrafenib, a BRAF inhibitor and Trametinib, a MEK inhibitor, are approved for adjuvant treatment.^5^

The pivotal trials of these drugs showed significant improvement in recurrence‐free survival compared to Ipilimumab (4‐year‐RFS: 51.7% vs. 41.2% in CheckMate 238) and placebo (5‐year‐RFS: 55.4% vs. 38.3% in KEYNOTE‐054 and 52% vs. 36% in COMBI‐AD).^6^, ^7^, ^8^, ^9^, ^10^, ^11^, ^12^ Retrospective real‐world studies have demonstrated the drugs' efficacy in clinical settings.^13^, ^14^, ^15^, ^16^

However, little is known about the effectiveness of a second course of adjuvant treatment in patients who progressed during or after their initial course of adjuvant melanoma treatment.

This retrospective study assesses RFS and adverse events in patients who underwent a second course of adjuvant melanoma therapy using Nivolumab, Pembrolizumab or Dabrafenib plus Trametinib after full surgical resection of their recurring disease.

Furthermore, overall survival (OS) and descriptive variables such as clinical, histological and molecular factors were investigated.

## METHODS

We retrospectively analysed 66 patients with stage III and IV melanoma who relapsed after previous adjuvant therapy with Nivolumab (NIV), Pembrolizumab (PEM) or Dabrafenib plus Trametinib (D + T) and subsequently underwent a second course of adjuvant treatment (Figure S6). In cooperation with skin cancer centres in Germany, Austria and Switzerland, we collected data between 01/2017 and 10/2021. Inclusion criteria were relapse under previous adjuvant therapy in stage III and IV, as well as complete resection before new adjuvant intervention. RFS was defined as the time from the start of therapy until relapse, death or exclusion for other causes. In patients without relapse, RFS was terminated at the last patient contact. Further analyses included overall survival, adverse events and subgroup analysis including clinical, histological and demographic factors. We used the Common Terminology Criteria for Adverse Events (CTCAE), version 5.0, as a guideline for the assessment of adverse events.

## STATISTICAL ANALYSIS

Statistical analyses were performed using SPSS version 27, as well as PRISM GraphPad. It included descriptive statistics, as well as survival analyses. The survival analyses were illustrated by Kaplan‐Meier curves, and comparisons were made using LogRank tests. The impact of select variables on RFS and OS was carried out using COX regression. The graphical representation of these results was done using forest plots with a 95% confidence interval.

## RESULTS

In the following analyses, patients were divided into PD‐1 (NIV/PEM) and BRAF + MEK (D + T) cohorts based on their second course of adjuvant melanoma therapy after recurrence during or after the first adjuvant treatment (RFS2).

### Patient characteristics

Sixty‐six patients from 22 centers in Germany, Austria and Switzerland were included. Thirty‐two patients received D + T as the second‐course adjuvant therapy after recurrence, 9 patients received PEM and 25 patients received NIV (Figure S6). Due to the small cohorts and no significant differences between NIV and PEM groups, they were combined into one group representing PD‐1 antibody‐treated patients. The median patient age was similar in PD‐1 and BRAF + MEK‐treated patient cohorts, with 56.5 (27–85) years and 56 (27–86) years, respectively. Within the total cohort, the male proportion of patients predominated with 33 male (50.0%) versus 26 female (39.4%) patients, whereby it should be noted that the sex of 7 patients was not determined (Table 1). Demographic parameters, tumour characteristics, whether primary or recurrent before new therapy, therapy characteristics such as length and time till the second course of adjuvant treatment, and AE, whether any or drug related, showed no significance comparing PD‐1 and BRAF + MEK‐treated patients (Table S1).

**TABLE 1:** Patient and first recurrence characteristics.

	PD‐1 (*N* = 34)	BRAF‐MEK (*N* = 32)
*Age*		
Median yrs. (range)	56.5 (27–85)	56 (27–86)
Male – No. (%)	19 (55.9)	14 (43.8)
Female – No. (%)	11 (32.4)	15 (46.9)
Not reported – No. (%)	4 (11.8)	3 (9.4)
*BMI*		
20–25	6 (17.6)	4 (12.5)
25–30	8 (23.5)	9 (28.1)
>30	8 (23.5)	5 (15.6)
Not reported	12 (35.3)	14 (43.8)
*Stage after 1. recurrence – No. (%)*		
IIIA	0 (0)	0 (0)
IIIB	7 (20.6)	5 (12.5)
IIIC	18 (52.9)	22 (68.8)
IIID	0 (0)	0 (0)
IV	9 (26.5)	6 (18.8)
*Type of 1. recurrence – No. (%)*		
Local recurrence	4 (11.8)	9 (28.1)
Lymph node metastasis only	11 (32.4)	12 (37.5)
Distant metastasis only	7 (20.6)	5 (15.6)
Distant + LN metastasis	2 (5.9)	7 (21.9)
Not reported	12 (35.3)	8 (25)
*No. of lymph node metastasis (%)*		
1	14 (41.2)	15 (46.9)
2–3	4 (11.8)	3 (9.4)
≥4	1 (2.9)	(12.5)
Not reported	14 (41.2)	10 (31.1)
*Organs affected by distant metastases – No. (%)*		
Skin	3 (8.8)	3 (9.4)
Lung	2 (5.9)	2 (6.3)
Other except brain	2 (5.9)	0 (0)
Brain	2 (5.9)	8 (25.0)
*V600 Mutation – No. (%)*		
Mutation	23 (67.6)	32 (100)
No Mutation	10 (29.4)	0 (0)
Not reported	1 (2.9)	0 (0)

### Primary tumour characteristics and treatment

Patients receiving either therapy showed an increased number of primary tumours with a thickness of more than 2 mm (PD‐1: 22 (64.7%), BRAF + MEK: 19 (56.6%)). There was no difference in the number of ulcerated tumours when comparing targeted agents or immuno‐oncology therapy‐treated cohorts. BRAF‐V600 mutations were present in 26 (74.6%) patients treated with PD‐1 antibodies and 32 (100%) patients treated with BRAF + MEK inhibitors. In terms of lymph node metastases (LNM) before first adjuvant therapy, clinically detectable LNM outnumbered occult LNM with 18 patients (52.9%) under PD‐1 and 15 patients (46.9%) under BRAF + MEK therapy. Laboratory parameters such as S100B and LDH were elevated in only a small percentage of patients in both groups. Most patients were in stage IIIC (PD‐1: 61.8% vs. BRAF + MEK: 65.6%) (Table S1). Prior to the first adjuvant treatment, sentinel lymph node‐biopsy (SLNB) was performed in 32.4% (PD‐1 Group) versus 40.6% (BRAF + MEK Group) patients, followed by total lymph node dissection (TLND) in 47.1% (PD‐1 group) and 40.6% (BRAF + MEK group). Duration of first adjuvant therapy was 3.5 (0–13) versus 5.0 (0–13) months in PD‐1 and BRAF + MEK‐treated patients respectively. Follow‐up covered 15.5 (3–53) and 25 (3–45) months (Table S2).

### Disease characteristics before second‐course adjuvant treatment

The median RFS after first adjuvant treatment was 7 (1–36) months in the PD‐1 and 17 (1–42) months in the BRAF + MEK cohort (Table S2). Stage IIIC also predominated after relapse (52.9% in PD‐1 vs. 68.8% in BRAF + MEK cohort), but the proportion of patients in stage IV increased markedly. Most patients had lymph node metastases (LNM; PD‐1: 32.4%; BRAF + MEK: 37.5%), sole distant metastasis (M) or the combination of LNM and M was found in 9 (26.8%) patients in the PD‐1 and 12 (37.5%) in the BRAF + MEK cohort, whereby distant metastases predominantly involved the skin (PD‐1: 8.8%; BRAF + MEK: 9.4%). Most patients showed singular metastases in different organs (PD‐1: 41.2%, BRAF + MEK 46.9%) (Table 1).

### Second adjuvant treatment and survival

Thirty‐four patients received PD‐1 and 32 BRAF + MEK as second adjuvant treatment after relapse and surgery. Initial adjuvant PD‐1 treatment was followed by a second course of BRAF + MEK inhibition in 63% and a second course of PD‐1 treatment in 37%. Initial adjuvant BRAF + MEK treatment was followed by a second course of BRAF + MEK inhibition in 6.3% and a second course of PD‐1 treatment in 93.8% of patients (Table 2).

**TABLE 2:** Second‐line adjuvant therapy.

	PD‐1 (*N* = 34)	BRAF + MEK (*N* = 32)
*Lymph node surgery – No. (%)*		
Lymph node excision (limited and/or TLND)	9 (26.5)	20 (62.3)
Excision of recurrent disease	19 (55.9)	10 (31.3)
Not reported	6 (17.6)	2 (6.3)
*First‐line adjuvant therapy – No. (%)*		
PD‐1 adjuvant	17 (50.0)	29 (90.6)
BRAF + MEK adjuvant	15 (44.1)	1 (3.2)
Not reported	2 (5.9)	2 (6.3)
*Second‐line adjuvant therapy – No. (%)*		
From PD‐1 to	17 (37.0)	29 (63.0)
From BRAF + MEK to	15 (93.8)	1 (6.3)
Radiation	11 (32.4)	7 (21.9)
*Blood results before second‐line adjuvant therapy – Median (range)*		
LDH	198 (136–786)	222 (128–686)
S100B	59 (22–2240)	60 (0–310)
NLR	1.9 (1.2–5.0)	2.63 (1.37–5.13)
Albumin	4.5 (4.0–4.9)	4.25 (3.7–5.7)
LDH ≥ ULN	22 (64.7)	11 (34.4)
S100 ≥ ULN	13 (38.2)	2 (6.3)
*Timing for second‐line adjuvant treatment – Median months (range)*		
Time till treatment start	1 (0–23)	1 (0–7)
Treatment duration	3.5 (0–13)	12 (1–14)
Follow‐up after treatment start	9.5 (0–29)	12 (0–29)

Prior to the second course of adjuvant therapy, all patients received full surgical resection including total lymph node dissection and metastasectomy. No increase in S100B or LDH parameters prevailed before renewed adjuvant therapy. The median time between excision and the start of new adjuvant therapy was 1 (0–23) month in the PD‐1 and 1 (0–7) month in the BRAF + MEK‐treated group. The duration of therapy was 3.5 (0–13) vs. 12^1^, ^2^, ^3^, ^4^, ^5^, ^6^, ^7^, ^8^, ^9^, ^10^, ^11^, ^12^, ^13^, ^14^ months, with 28 patients completing PD‐1 and 27 patients completing BRAF + MEK treatment. The follow‐up period was 9.5 (0–29) months for PD‐1 and 12 (0–29) months for BRAF + MEK (Table 2).

Eighteen (52.9%) patients recurred under PD‐1 treatment and 10 (31.3) under BRAF + MEK. The median time to recurrence was 11 (0–32) months using PD‐1 antibodies, versus 17 (1–42) using BRAF + MEK inhibition. Lymph node metastases were found in 7 (20.6%) patients receiving PD‐1 and 3 (9.4%) patients receiving BRAF + MEK treatment. Distant metastases were found at similar rates in both groups: 8 (23.6%) versus 7 (22.0%) patients (Table 3). It should be emphasized that patients who received SLNB only did not show a significantly increased number of lymph node metastases in case of recurrence under renewed adjuvant therapy compared to those who initially received SLNB ± TLND (Tables 1 and S3).

**TABLE 3:** Treatment failures of second‐line adjuvant therapy.

	PD‐1 (*N* = 34)	BRAF‐MEK (*N* = 32)
*End of therapy due to – No. (%)*		
Adverse events	2 (5.9)	5 (15.6)
Melanoma recurrence	18 (52.9)	10 (31.3)
Local recurrence	2 (5.9)	1 (3.2)
Lymph node metastasis only	7 (20.6)	3 (9.4)
Distant metastasis only	4 (11.8)	6 (18.8)
Distant + LN metastasis	4 (11.8)	1 (3.2)
Not reported	2 (5.9)	0 (0)
Time to recurrence – Median months (range)	11 (0–32)	17 (1–42)
*Therapy of recurrence – no. (%)*		
Surgery	9 (60.0)	6 (60.0)
Radiation therapy	5 (33.3)	3 (30.0)
Systemic therapy	12 (80.0)	7 (70.0)
Not reported	3 (20.0)	0 (0)

**FIGURE 1:**
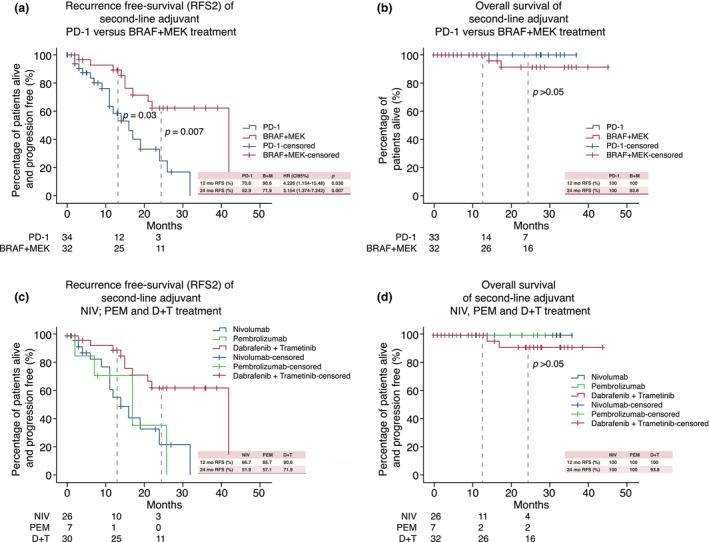
RFS and OS of second‐line adjuvant treatment. (a–d) Kaplan Meier curves of recurrence‐free survival (RFS2) and overall survival (OS) after 12 and 24‐months. Statistical differences were assessed using COX regression. (a) Significant differences in RFS2 comparing second‐line adjuvant PD‐1 and BRAF + MEK treatment. (b) No differences in OS were observed. (c) RFS2 and (d) OS for the individual adjuvant drugs. D + T, Dabrafenib and Trametinib; HR, hazard ratio; NIV, nivolumab; PEM, pembrolizumab.

The recurrence‐free survival of the second‐line adjuvant treatment (RFS2) was analysed after 12 and 24 months. At the 12‐month mark, RFS2 was significantly shorter in patients receiving PD‐1 therapy versus BRAF + MEK therapy: 70.6% versus 90.6% (HR 4.226 [95% CI 1.154–15.48]; *p* = 0.030). This difference remained significant at the 24‐month follow‐up (HR 3.154 [95% CI 1.374–7.242]; *p* = 0.007) (Figure 1). Real‐world RFS1 using modern adjuvant therapy has already been described previously. Compared to RFS1, 12‐month RFS2 was similar for both adjuvant PD‐1 blockade and BRAF + MEK inhibition. However, data revealed a drop in RFS2 with 52.9% (RFS2) compared to 67.9% (RFS1) at 24 months in the adjuvant PD‐1 treatment group.^16^

There was no significant difference in overall survival between the two cohorts, with 100% using PD‐1 and 93.8% using BRAF + MEK (*p* > 0.5) (Figure 1b,d).

### Subgroup analyses in BRAF V600 mutant patients

Limiting analyses to BRAF (V600) mutant tumours, 23 patients receiving initial PD‐1 therapy and 32 patients receiving initial BRAF + MEK therapy could be assessed.

**FIGURE 2:**
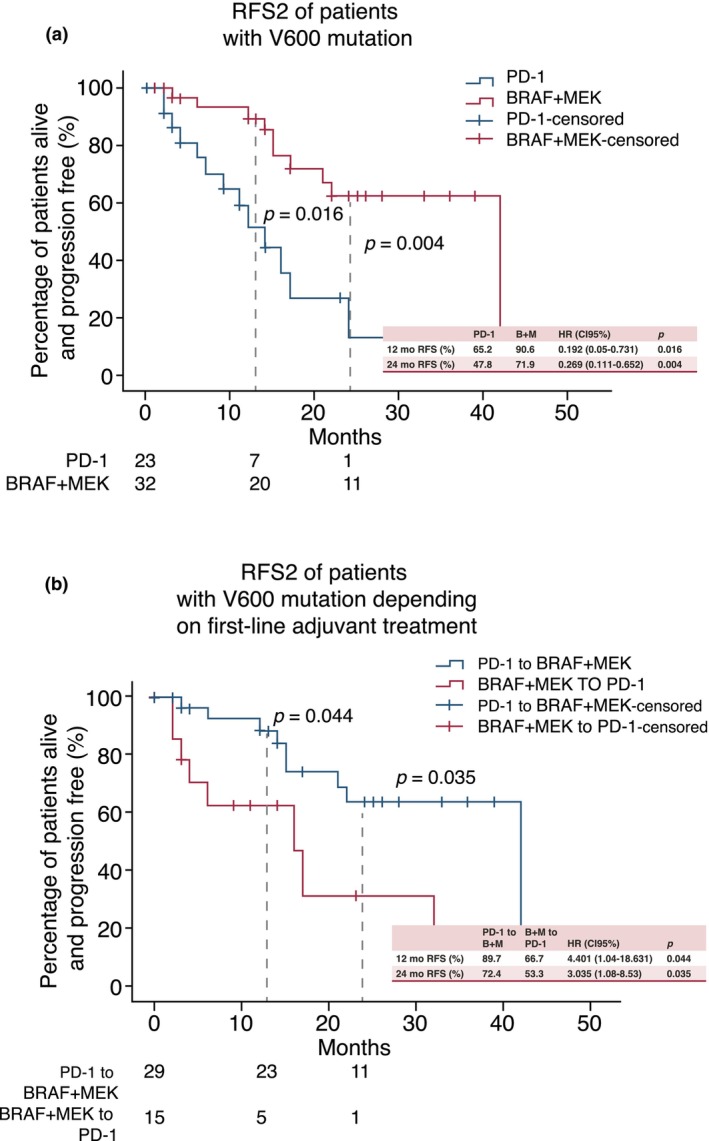
RFS of patients with V600 mutation. Kaplan Meier curves of recurrence‐free survival (RFS2) and overall survival (OS) after 12 and 24‐months for BRAF(V600) mutant melanoma patients. Statistical differences were assessed using COX regression. (a) Significant differences in RFS2 comparing second‐line adjuvant PD‐1 and BRAF + MEK treatment. (b) Significant differences in RFS2 in patients receiving first‐line adjuvant anti PD‐1 treatment followed by BRAF + MEK blockade compared to patients receiving first‐line BRAF + MEK blockade followed by anti PD‐1 treatment. HR, hazard ratio.

Regardless of initial adjuvant treatment, the second course of adjuvant BRAF + MEK treatment resulted in a significantly better RFS2 than PD‐1 treatment (HR 0.192 (0.05–0.731), *p* = 0.016) (Figure 2a). Furthermore, the overall recurrence‐free survival, consisting of the RFS from first plus second adjuvant therapy, was assessed. In this cohort, there was a superiority in total RFS in patients who initially received PD‐1 antibodies compared to BRAF + MEK inhibitors (HR 2.546 (1.042–6.223), *p* = 0.04) (Figure S4).

There was a positive trend for improved RFS in patients who received a class switch. It needs to be pointed out that “no class switch” mainly refers to patients who received 2 courses of adjuvant PD‐1 treatment, since only one patient with initial BRAF + MEK adjuvant treatment received a second course of adjuvant BRAF + MEK treatment (Figures S1 and S2).

BRAF V600 mutant patients who were first treated with PD‐1 antibodies and received a second course of adjuvant BRAF + MEK treatment had better 12‐month RFS2 compared to patients being switched from initial BRAF + MEK to second‐course adjuvant PD‐1 treatment (HR 4.401 (1.04–18.63), *p* = 0.044) (Figure 2b).

There was no difference in RFS for second‐course adjuvant PD‐1 treatment in BRAF V600 mutant versus BRAF wild‐type melanoma (Figure S3).

### Adverse events (AE)

Data from NIV‐ and PEM‐treated patients were combined due to the small cohort. Compared to data from the pivotal trials, both therapies, PD‐1 and BRAF + MEK, showed no new safety signals.^6^, ^7^, ^8^

Under PD‐1 treatment, 19 (55.9%) patients experienced any AE, compared to 23 (71.9%) under BRAF + MEK. Two (5.9%) patients experienced grade III or higher AE using PD‐1, compared with 4 (12.5%) using BRAF + MEK (Table S4).

Drug‐related adverse events (drAE) were found in 14 (44.1%) patients with grade III and higher in 4 (11.9%) under PD‐1 treatment. Leading drAEs were rash (8.8%) and diarrhoea (6.9%). Reported grade III and higher drAEs were pneumonitis, hepatitis, meningitis and blood count changes with elevated amylase and lipase. Under BRAF + MEK therapy, drAE were observed in 16 (50.0%) patients, with grade III and higher observed in 2 (6.3%). Leading drAEs were pyrexia (18.75%) and rash (9.3%). Reported grade III and higher drAEs were pneumonitis and laboratory CK elevation (Table S4). AEs leading to treatment discontinuation occurred in 5.9% (PD‐1) and 15.6% (BRAF + MEK) of patients (Table 3). No death due to AEs was reported.

## DISCUSSION

This study assessed recurrence‐free and overall survival of patients receiving a second course of adjuvant therapy after relapse and surgery under or after a previous adjuvant regimen using PD‐1 and BRAF + MEK Inhibitors.

Indirect comparison to pivotal clinical trials revealed that there was no significant decrease in the efficacy of second‐line adjuvant treatment in 12‐month RFS when adjuvant therapy was repeated after recurrence: CheckMate238 (NIV) showed a 12‐month RFS of 70.5% compared to 66.7% when NIV was re‐administered in this study. Similar results were found for second‐line PEM (85.7% vs. 75.5% in KEYNOTE‐054), and for BRAF + MEK blockade (90.6% vs. 88% in Combi‐AD).^6^, ^7^, ^8^ At 24‐month, RFS dropped markedly: 51.9% with NIV, 71.4% with PEM and 72.2% with D + T. Combining the results of both PD‐1 antibodies, RFS2 at 24 months was 55%. If one takes into account the fact that only a small number of patients were still receiving therapy at the end of the observation period and that most relapses occurred after the end of second‐line adjuvant treatment, the question arises as to whether systemic therapy should be continued for more than 12 months in these patients. Unfortunately, no data are currently available to answer this question.

Furthermore, the 12‐ and 24‐month overall survival rates were excellent for both PD‐1 and BRAF‐MEK therapies, highlighting the high efficacy of both treatment regimens.

Data from this study suggest that the choice of first‐line adjuvant therapy might be important in patients who have recurrent disease and melanoma can be fully resected again. Since BRAF + MEK inhibitors can only be used in BRAF(V600) mutant patients, analyses were restricted to this group of patients. In this study, BRAF + MEK blockade was superior to second‐line adjuvant PD‐1 treatment in BRAF(V600E) mutant melanoma (HR 0.192 (0.05–0.731), *p* = 0.016) (Figure 2a). These findings are supported by recent reports where the adjuvant second‐line treatment with BRAF + MEK versus PD‐1 inhibition was convincing, with an RFS2 of 41 versus 6 months, regardless of the adjuvant first‐line regimen.^17^

Additionally, comparing patients treated with PD‐1 inhibition as first‐line adjuvant therapy tended to have a better outcome at 12 months and significantly better outcome at 24 months compared to those initially treated with BRAF + MEK (HR 2.546 (1.042–6.223), *p* = 0.04) (Figure S4). Data further suggest that switching the treatment regimen might be beneficial in cases where a second course of adjuvant therapy is considered after surgery. Switching from PD‐1 to BRAF + MEK was significantly better than switching from BRAF + MEK to PD‐1 (HR 4.401 (1.04–18.63), *p* = 0.044) (Figure 2b). However, it must be noted that only one patient received first‐ and second‐line adjuvant BRAF + MEK treatment. Accordingly, the group of patients with no class switch primarily refers to patients who were treated with adjuvant PD‐1 antibodies twice. Consequently, although the superiority of BRAF + MEK after a class switch was evident, no conclusion can be drawn as to whether the continuation of adjuvant BRAF + MEK blockade after relapse and surgery may be effective.

Furthermore, the question arises as to why the switch from BRAF + MEK to PD‐1 inhibition performs significantly worse in this study. One possibility would be an altered tumour microenvironment through, for example, changes in macrophages, lymphocytes, altered PD‐(L)1 expression or selected immune‐cell compositions as described in stage IV melanoma.^18^, ^19^, ^20^ The molecular basis underlying the observed changes in this cohort needs to be investigated in future studies.

Variables potentially affecting outcome, including the number and type of metastases, the extent of surgery, drug‐related adverse events (drAE), LDH and S100B did not aid in stratifying for improved RFS (Figure S5A,B).

Safety data revealed that 4 (11.8%) patients under PD‐1 and 2 (6.3%) patients under BRAF + MEK experienced drAE grade ≥3 (Table S4). All adverse events greater than or equal to grade 3 occurred within the first 12 months of second‐line adjuvant treatment. It must be taken into account that it is impossible to fully distinguish delayed drAE caused by first‐line adjuvant therapy from drAE after renewed adjuvant treatment. Yet, drAEs of second‐line adjuvant therapy occurred on average more than 3 months after restarting therapy.

This study has strengths and limitations. The small cohort of 66 patients and the retrospective nature of the study must be considered when interpreting data. Yet, little is known about second‐line adjuvant therapy and how the choice of first‐line treatment might affect overall outcome. Similar to the treatment of stage IV disease, data suggest that first‐line adjuvant checkpoint inhibition followed by second‐line adjuvant BRAF + MEK blockade might yield better overall RFS in a select BRAF(V600E) mutant patient cohort.^20^

## CONCLUSIONS

In summary, when second‐line adjuvant therapy is considered, data from this study suggest that a class switch, particularly PD‐1 to BRAF + MEK inhibition, results in improved RFS and potentially also improved OS.

## AUTHOR CONTRIBUTIONS

KS and CP designed and executed the study and wrote the manuscript. SK and CP performed the statistical analyses. All other coauthors collected and contributed patient data for the study and proofread the manuscript.

## FUNDING INFORMATION

This work was supported by a grant from the family of Prof. Engelbert Dockner.

## CONFLICT OF INTEREST STATEMENT

Alexander Thiem received consulting fees and participation on a Data Safety Monitoring Board or Advisory Board from Bristol‐Myers Squibb (BMS) and Merck Sharp & Dohme (MSD); received payment or honoraria for lectures, presentations, speakers bureaus, manuscript writing or educational events from BMS, GlaxoSmithKline (GSK), Kyowa Kirin, Recordati Rare Diseases (RRD) and Sanofi; received support for attending meetings and/or travel from BMS, MSD, Novartis and Pierre Fabre Pharmac; and participated in a data safety monitoring board or advisory board for BMS and MSD. Andreas Dominik Braun received consulting fees and participated in a Data Safety Monitoring Board or Advisory Board from and for MSD Sharp & Dohme GmbH and received support for attending meetings and/or travel from Novartis. Miriam Mengoni received consulting fees from MSD Sharp & Dohme GmbH and BMS; received payment or honoraria for lectures, presentations, speakers bureaus, manuscript writing or educational events from MSD Sharp & Dohme GmbH, Novartis, BMS, Stemline Therapeutics, Kyowa Kirin and Pierre Fabre; received support for attending meetings and/or travel from Pierre Fabre, Sun Pharma and LEO Pharma; and participated in a Data Safety Monitoring Board or Advisory Board for MSD Sharp & Dohme GmbH and BMS. Reinhard Dummer received consulting fees from Amgen, BMS, MSD, Novartis, Pierre Fabre, Roche, Sun Pharma, Takeda, Sanofi, Caralym, Second‐Genome, Regeneron, Alligator, T3 Pharma, MaxiVAX SA, Pfizer, Simcere and touchIME; and received payment or honoraria for lectures, presentations, speakers bureaus and manuscript writing or educational events from Amgen, BMS, MSD, Novartis, Pierre Fabre, Roche, Sun Pharma, Takeda, Sanofi, Caralym, Second‐Genome, Regeneron, T3 Pharma, MaxiVAX SA, Pfizer and Simcere. Lara Valeska Maul received support for attending meetings and/or travel from Almirall, Amgen, BMS, Incyte, MSD, Novartis, Pierre Fabre and Roche and Sanofi; participated in a Data Safety Monitoring Board or Advisory Board for Almirall, Amgen, BMS, Incyte, MSD, Novartis, Pierre Fabre and Roche and Sanofi; and received grants or contracts from Research Fund University Basel, Switzerland; Voluntary Academic Society, Basel, Switzerland; and ProPatient Foundation, University Hospital Basel, Switzerland. Wiebke K. Peitsch received payment or honoraria for lectures, presentations, speakers bureaus, manuscript writing or educational events from BMS, MSD, Novartis, Pfizer and Roche; received support for attending meetings and/or travel from BMS, MSD, Novartis, Pfizer, Roche and Sanofi; played a leadership or fiduciary role in other board, society, committee or advocacy group; received grants or contracts, paid or unpaid, from Berliner Dermatologische Gesellschaft, Sun Pharma and Immunocore. Oana‐Diana Persa received payment or honoraria for lectures, presentations, speakers bureaus, manuscript writing or educational events from MSD; received support for attending meetings and/or travel from Kyowa Kirin, Pierre Fabre, Sanofi, Sun Pharma; and participated in a Data Safety Monitoring Board or Advisory Board for BMS, Sanofi. Christian Posch received consulting fees from Novartis; received payment or honoraria for lectures, presentations, speakers bureaus, manuscript writing or educational events from MSD, BMS, Novartis, Pierre Fabre, PelPharma, Leo Pharma, Almirall, AbbVie, Celgene, Sanofi, Pfizer, Eli Lilly, DSD, Janssen, Astra Zeneca, MERCK and Takeda; received support for attending meetings and/or travel from MSD, BMS, Novartis, Pierre Fabre, PelPharma, Leo Pharma, Almirall, AbbVie, Sanofi, Pfizer and Janssen; received grants or contracts from Almirall. Steffen Emmert received payment or honoraria for lectures, presentations, speakers bureaus, manuscript writing or educational events from Amgen, BMS, MSD, Novartis, LEO, Sanofi, Pierre Fabre, Pfizer, Janssen, Abbvie, UCB, Almirall, Galderma, Mayne Genzyme Corporation, Malinckrodt, SUN Pharma, Oncobeta, SolGel, RheaCell, Teion and CINOGY; received support for attending meetings and/or travel from Amgen, BMS, MSD, Sanofi, Pierre Fabre, Therakos and Cinogy; participated in a Data Safety Monitoring Board or Advisory Board for Rheacell and SolGel; played a leadership or fiduciary role in another board, society, committee or advocacy group; received grants or contracts from the National Center for Plasma Medicine, North German Dermatological Society and Leibniz Research Network ‘Health Technologies’. Frank Meiß received consulting fees from Pierre Fabre Pharma GmbH, Novartis Pharma GmbH, Sun Pharmaceuticals Industries Europe B.V. and BMS GmbH & Co. KGaA; received payment or honoraria for lectures, presentations, speaker bureaus, manuscript writing or educational events from Pierre Fabre Pharma GmbH, Novartis Pharma GmbH, Sun Pharmaceuticals Industries Europe B.V. and BMS GmbH & Co. KGaA; received support for attending meetings and/or travel from Novartis Pharma GmbH, Sun Pharmaceuticals Industries Europe B.V. and Pierre Fabre Pharma GmbH. Van Anh Nguyen received payment or honoraria for lectures, presentations, speaker bureaus, manuscript writing or educational events from BMS, MSD, Novartis and Pierre Fabre; received support for attending meetings and/or travel from BMS, MSD and Pierre Fabre; and participated in a Data Safety Monitoring Board or Advisory Board for BMS, MSD, Novartis and Pierre Fabre. Marina Wanner received payment or honoraria for lectures, presentations, speaker bureaus, manuscript writing or educational events from BMS and received support for attending meetings and/or travel from Sanofi, Abbvie and BMS. Peter Kölbinger received consulting fees from BMS, Merck Sharp and Dohme, Novartis, Pierre Fabre and Sanofi Aventis; received payment or honoraria for lectures, presentations, speakers bureaus, manuscript writing or educational events from BMS, Merck Sharp and Dohme, Novartis, Pierre Fabre and Sanofi Aventis; received support for attending meetings and/or travel from Pierre Fabre, Merck Sharp and Dome. Carmen Loquai received consulting fees from BMS, MSD, Roche, Novartis, Sun Pharma, Kyowa Kirin, Sanofi, Biontech, Almirall Hermal, Lilly and Regeneron; received payment or honoraria for lectures, presentations, speakers bureaus, manuscript writing or educational events from BMS, MSD, Roche, Novartis, Sun Pharma, Kyowa Kirin, Sanofi, Biontech, Almirall Hermal, Lilly and Regeneron; and received support for attending meetings and/or travel from BMS, MSD, Roche, Novartis, Sun Pharma, Kyowa Kirin, Sanofi, Biontech, Almirall Hermal, Lilly and Regeneron. Erika Richtig received consulting fees from Amgen, Bayer, BMS, Delcath, MSD, Merck, Novartis and Pierre Fabre; received payment or honoraria for lectures, presentations, speakers bureaus, manuscript writing or educational events from BMS, Delcath, MSD, Merck, Novartis and Pierre Fabre; received support for attending meetings and/or travel from Amgen, BMS, Merck Sharp 6 Dohme, Merck, Novartis, Pierre Fabre, Sanofi, Roche; participated in a Data Safety Monitoring Board or Advisory Board from BMS, MSD, Novartis, Pierre Fabre; played a leadership or fiduciary role in other boards, societies, committees or advocacy groups; received grants or contracts—paid or unpaid—from Austrian Cancer Aid/Styra, Arbeitsgruppe Melanom Dermatoonkologie (AMDO); stock or stock options from Roche Pharma AG; and others from Amgen, BMS, Delcath, Incyte, MSD, Merck, Novartis, Pfizer, Regeneron, Roche and Sanofi. Kai Christian Klespe received payment or honoraria for lectures, presentations, speakers bureaus, manuscript writing or educational events from BMS and RRD; received support for attending meetings and/or travel from Novartis, Sun Pharma, Kyowa Kirin and Pierre Fabre; participated in a Data Safety Monitoring Board or Advisory Board from Regeneron and Novartis; received grants or contracts from, payment for expert testimony, Vetter Pharma. The authors Katharina Schumann, Tilo Biedermann, Johanna Mangana, Magdalena Hoellwerth, Cornelia Mauch, Klemens Rappersberger, Ulrike Schultheis, Veronika Zwenderowski, Sevil Böger and Wolfgang Harth have nothing to report.

## ETHICAL APPROVAL

The study was approved by the ethical committee of the Technical University of Munich and in all participating centres.

## ETHICS STATEMENT

This trial is a retrospective data analysis of 22 data sets from Germany, Austria and Switzerland. Data were pseudonymized and does not contain information or images that could identify individuals. According to ethical committee regulations, patient consent was voided.

## Supporting information


Figure S1.

Figure S2.

Figure S3.

Figure S4.

Figure S5.

Figure S6.

Table S1.

Table S2.

Table S3.

Table S4.

## Data Availability

The data that support the findings of this study are available from the corresponding author upon reasonable request.
