# Supplementary material for: Switching PD‐1 to BRAF + MEK inhibition improves recurrence‐free survival in patients receiving a second course of adjuvant melanoma therapy
Source: J Eur Acad Dermatol Venereol. 2025 May 7;39(11):1987–96. doi: 10.1111/jdv.20708 (PMC12553123; doi:10.1111/jdv.20708)
Supplement: Supplementary file 1 — Figure S1. [file JDV-39-1987-s004.docx]

Figure 1 **Class Switch – Independent of V600 Mutation**

**Figure 1A-C:**

Survival analysis of recurrence free survival (RFS2) using Kaplan Meier curves and Cox regression.

**1A: Comparison of RFS2 in relation to switching between individual adjuvants. 1B: Comparison of RFS2 in relation to switching between substance classes. 1C: Comparison of RFS2 between class switching versus no class switching.**
