# Supplementary material for: Switching PD‐1 to BRAF + MEK inhibition improves recurrence‐free survival in patients receiving a second course of adjuvant melanoma therapy
Source: J Eur Acad Dermatol Venereol. 2025 May 7;39(11):1987–96. doi: 10.1111/jdv.20708 (PMC12553123; doi:10.1111/jdv.20708)
Supplement: Supplementary file 2 — Figure S2. [file JDV-39-1987-s008.docx]

Figure 2 **RFS2 in V600 Mutated Patients: Class Switch vs. No Class Switch**

**Figure 2:**

Kaplan Meier curves of recurrence-free survival (RFS2) after 12 and 24-months. Statistical differences were assessed using COX regression**.** When comparing class switch versus no class switch, regardless of the individual substance classes, there is no significant difference with regard to recurrence free survival (RFS2)
