# Supplementary material for: Switching PD‐1 to BRAF + MEK inhibition improves recurrence‐free survival in patients receiving a second course of adjuvant melanoma therapy
Source: J Eur Acad Dermatol Venereol. 2025 May 7;39(11):1987–96. doi: 10.1111/jdv.20708 (PMC12553123; doi:10.1111/jdv.20708)
Supplement: Supplementary file 3 — Figure S3. [file JDV-39-1987-s006.docx]

Figure 3 **Second line anti PD-1 treatment after recurrence following adjuvant PD-1 treatment in BRAF(V600) mutant versus BRAF(V600) wildtype melanoma patients**

**Figure 3:**

Kaplan Meier curves of recurrence-free survival (RFS2) after 12 and 24-months. Statistical differences were assessed using COX regression. There was no significant difference between V600 mutation versus no mutation.
