# Supplementary material for: Switching PD‐1 to BRAF + MEK inhibition improves recurrence‐free survival in patients receiving a second course of adjuvant melanoma therapy
Source: J Eur Acad Dermatol Venereol. 2025 May 7;39(11):1987–96. doi: 10.1111/jdv.20708 (PMC12553123; doi:10.1111/jdv.20708)
Supplement: Supplementary file 4 — Figure S4. [file JDV-39-1987-s002.docx]

Figure 4 **RFS in patients under V600 mutation regarding to first adjuvant treatment**

**Figure 4:**

Kaplan Meier curves of total recurrence-free survival (RFS1 + RFS2). Statistical differences were assessed using COX regression. There was a superiority in total RFS in patients who initially received PD-1 antibodies compared to BRAF+MEK inhibitors (HR 2.546 (1.042-6.223), p=0.04).
