# Supplementary material for: Switching PD‐1 to BRAF + MEK inhibition improves recurrence‐free survival in patients receiving a second course of adjuvant melanoma therapy
Source: J Eur Acad Dermatol Venereol. 2025 May 7;39(11):1987–96. doi: 10.1111/jdv.20708 (PMC12553123; doi:10.1111/jdv.20708)
Supplement: Supplementary file 5 — Figure S5. [file JDV-39-1987-s010.docx]

Figure 5 **Variables affecting adjuvant treatment**

**Figure 5A+B:** Individual variables and their influence on adjuvant therapy with BRAF+MEK or PD-1 inhibitors were investigated. The comparison was carried out using COX regression. A forest plot was created for visualisation. A: No variable with a significant influence on recurrence free survival (RFS2) was found under BRAF+MEK therapy. B: No variable with a significant influence on recurrence free survival (RFS2) was found under PD-1 therapy.
