# Supplementary material for: Switching PD‐1 to BRAF + MEK inhibition improves recurrence‐free survival in patients receiving a second course of adjuvant melanoma therapy
Source: J Eur Acad Dermatol Venereol. 2025 May 7;39(11):1987–96. doi: 10.1111/jdv.20708 (PMC12553123; doi:10.1111/jdv.20708)
Supplement: Supplementary file 6 — Figure S6. [file JDV-39-1987-s009.docx]

Figure 6 – Flow Chart
