# Supplementary material for: Switching PD‐1 to BRAF + MEK inhibition improves recurrence‐free survival in patients receiving a second course of adjuvant melanoma therapy
Source: J Eur Acad Dermatol Venereol. 2025 May 7;39(11):1987–96. doi: 10.1111/jdv.20708 (PMC12553123; doi:10.1111/jdv.20708)
Supplement: Supplementary file 7 — Table S1. [file JDV-39-1987-s005.docx]

Table 1 **Primary Tumor Characteristics**

|  | **PD-1**  **(N=34)** | | | **BRAF+MEK**  **(N=32)** |
| --- | --- | --- | --- | --- |
| **Type of Tumor - No. (%)** | | | | |
| **SSM** | 7 (20.6) | | | 10 (31.3) |
| **NM** | 17 (50.0) | | | 9 (28.1) |
| **Other** | 7 (20.6) | | | 3 (9.4) |
| **Not reported** | 3 (8.8) | | | 7 (21.9) |
| **Thickness (mm) – No. (%)** | | | | |
| **< 1.0** | 4 (11.8) | | | 5 (15.6) |
| **1.01 – 2.0** | 4 (11.8) | | | 6 (18.8) |
| **2.01 – 4.0** | 14 (41.2) | | | 7 (21.9) |
| **> 4.01** | 8 (23.5) | | | 11 (34.4) |
| **Not reported** | 4 (11.8) | | | 3 (9.4) |
| **Ulceration - No. (%)** | | | | |
| **Yes** | 15 (44.1) | | | 15 (53.1) |
| **No** | 14 (41.2) | | | 13 (40.6) |
| **Not reported** | 5 (14.7) | | | 2 (6.3) |
| **Mutation - No. (%)** | | | | |
| **BRAF V600** | 26 (74.6) | | | 31 (96,9) |
| **Mutation not reported** | 0 (0) | | | 0 (0) |
| **Lymphatic disease* - No. (%)** | | | | |
| **≤ 1 pos. lymph nodes** | 21 (61.8) | | | 27 (84.4) |
| **2-3 pos. lymph nodes** | 7 (20.6) | | | 2 (6.3) |
| **≥ 4 pos. lymph nodes** | 6 (17.6) | | | 2 (6.3) |
| **clinically occult** | 7 (20.6) | | | 10 (31.3) |
| **clinically detectable** | 18 (52.9) | | | 15 (46.9) |
| **extra capsular** | 5 (14.7) | | | 6 (6.3) |
| **In transit / satellite metastasis** | 10 (29.4) | | | 12 (37.5) |
| **Not reported** | 1 (2.9) | | | 1 (3.1) |
| **Stage^#^ - No. (%)** | | | | |
| **IIIA** | 1 (2.9) | | | 1 (3.1) |
| **IIIB** | 6 (17.6) | | | 7 (21.9) |
| **IIIC** | 21 (61.8) | | | 21 (65.6) |
| **IIID** | 0 (0) | | | 0 (0) |
| **IV** | 2 (5.9) | | | 2 (6.3) |
| **Blood results before therapy - Median* (range)** | | | | |
| **LDH** | | 207.5 (121-358) | 216 (121-358) | |
| **S100B** | | 45 (0-350) | 58 (0-120) | |
| **NLR** | | 2.2 (0.97-8.68) | 2.21 (0.98-8.68) | |
| **LDH > ULN** | | 6 (17.6) | 10 (31.3) | |
| **S100 > ULN** | | 2 (5.9) | 1 (3.1) | |

*multiple entries possible

#according to AJCC 8th Edition

All laboratory values were collected +/-7d of start of therapy and were available for >70% of patients
