# Supplementary material for: Switching PD‐1 to BRAF + MEK inhibition improves recurrence‐free survival in patients receiving a second course of adjuvant melanoma therapy
Source: J Eur Acad Dermatol Venereol. 2025 May 7;39(11):1987–96. doi: 10.1111/jdv.20708 (PMC12553123; doi:10.1111/jdv.20708)
Supplement: Supplementary file 8 — Table S2. [file JDV-39-1987-s003.docx]

Table 2 **Primary Tumor Treatment**

|  | **PD-1**  **(N=34)** | | **BRAF+MEK**  **(N=32)** |
| --- | --- | --- | --- |
| **Lymph node surgery - No. (%)** | | | |
| **SLNB only** | 11 (32.4) | | 13 (40.6) |
| **TLND** | 16 (47.1) | | 13 (40.6) |
| **Not reported** | 7 (20.6) | | 6 (18.8) |
| **Timing – Median in months (range)** | | | |
| **Time till adjuvant treatment** | | 1.5 (0-12) | 1 (0.7) |
| **Treatment duration** | | 3.5 (0-13) | 5 (0-13) |
| **FU after treatment start** | | 15.5 (3-53) | 25 (3-45) |
| **RFS1** | | 7 (1-36) | 17 (1-42) |
| **First adjuvant treatment – No. (%)** | | | |
| **PD-1** | 17 (50.0) | | 29 (90.6) |
| **BRAF+MEK** | 15 (44.1) | | 1 (3.13) |

* All laboratory values were collected +/-7d of start of therapy and were available for >70% of patients

SLNB – sentinel lymph node dissection. TLND – complete lymph node dissection. LDH – lactat dehydrogenase. NLR – neutrophil to lymphocyte ratio. ULN – upper limit of the norm. FU – follow up.
