# Supplementary material for: Switching PD‐1 to BRAF + MEK inhibition improves recurrence‐free survival in patients receiving a second course of adjuvant melanoma therapy
Source: J Eur Acad Dermatol Venereol. 2025 May 7;39(11):1987–96. doi: 10.1111/jdv.20708 (PMC12553123; doi:10.1111/jdv.20708)
Supplement: Supplementary file 9 — Table S3. [file JDV-39-1987-s001.docx]

Table 3 **Type of lymph node extirpation and type of metastases in case of recurrence**

|  | **PD-1**  **(N=34)** | **BRAF-MEK**  **(N=32)** |
| --- | --- | --- |
|  | | |
| **TLND and LNM only** | 3 (8.8) | 3 (9.4) |
| **TLND and MM only** | 2 (5.9) | 2 (6.3) |
| **TLND and LNM + MM** | 3 (8.8) | 0 (0) |
| **SLNB and LNM only** | 3 (8.8) | 1 (3.1) |
| **SLNB and MM only** | 1 (2.9) | 1 (3.1) |
| **SLNB and LNM + MM** | 1 (2.9) | 0 (0) |
| **Initial treatment unknown** | 2 (5.9) | 3 (9.4) |
