# Supplementary material for: Switching PD‐1 to BRAF + MEK inhibition improves recurrence‐free survival in patients receiving a second course of adjuvant melanoma therapy
Source: J Eur Acad Dermatol Venereol. 2025 May 7;39(11):1987–96. doi: 10.1111/jdv.20708 (PMC12553123; doi:10.1111/jdv.20708)
Supplement: Supplementary file 10 — Table S4. [file JDV-39-1987-s007.docx]

**Table
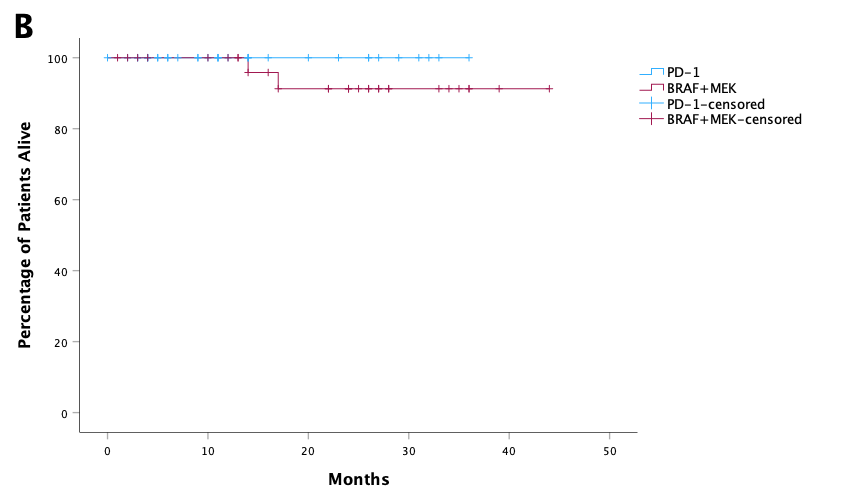

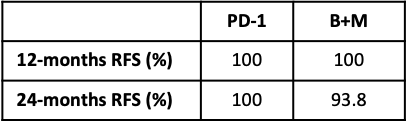

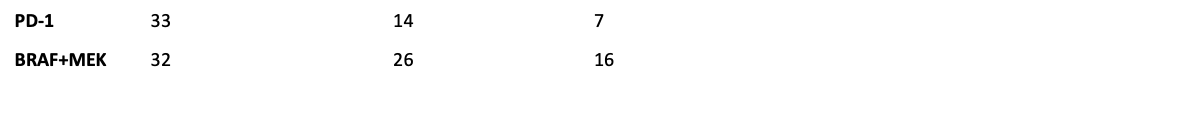

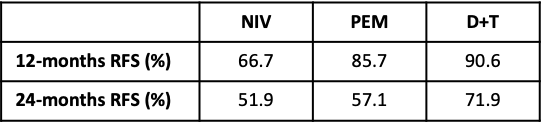

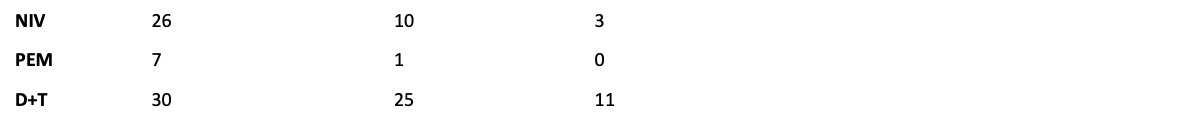

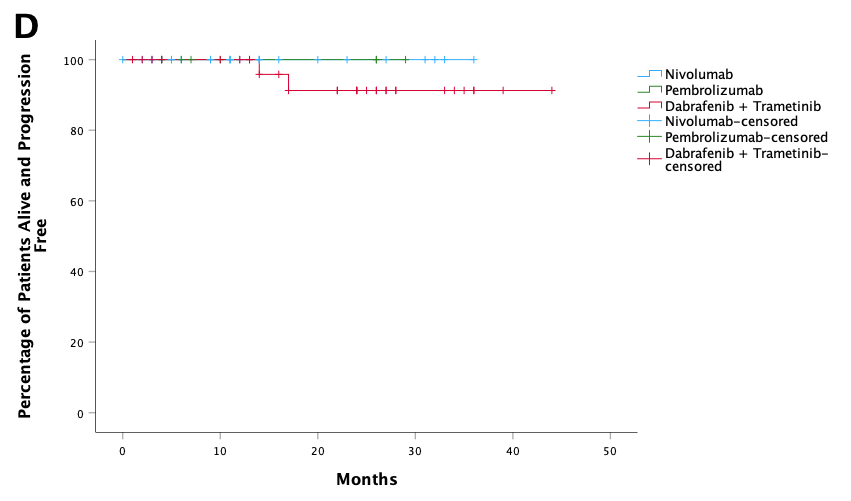

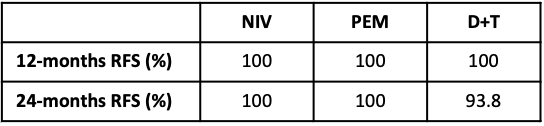

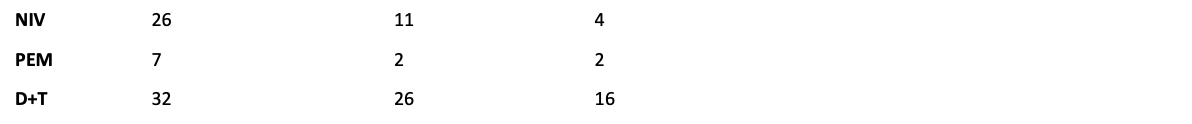
4 Adverse Events**

p > 0.05

**Overall Survial of second-line adjuvant PD-1 versus BRAF+MEK treatment**

p > 0.05

**Overall Survival of adjuvant NIV, PEM and D+T treatment**

**Figure 1 A-D**

Kaplan Meier curves of recurrence-free survival (RFS2) and overall survival (OS) after 12 and 24-months. Statistical differences were assessed using COX regression. **A.** Significant differences in RFS2 comparing second-line adjuvant PD-1 and BRAF+MEK treatment. **B.** No differences in OS were observed. **C.** RFS2 and **D.** OS for the individual adjuvant drugs.

HR – hazard ratio; NIV – nivolumab; PEM – pembrolizumab; D+T – Dabrafenib and Trametinib;

| **Adverse Events** | | **PD-1**  **(N=34)** | | | **BRAF+MEK**  **(N=32)** | |
| --- | --- | --- | --- | --- | --- | --- |
|  | **Number of patients** | | **Grade ≥ 3** | **Number of patients** | | **Grade ≥ 3** |
| **Total No.** | 34 | |  | 32 | |  |
| **Any adverse event (AE)** | 19 (55.9) | | 2 (5.9) | 23 (71,9) | | 4 (12.5) |
| **Not reported** | 0 (0) | | 0 (0) | 0 (0) | | 3 (9.3) |
| **Drug related AE** | 15 (44.1) | | 4 (11.8) | 16 (50) | | 2 (6.3) |
| **Not reported** | 0 (0) | | 0 (0) | 0 (0) | | 2 (6.3) |
|  |  | |  |  | |  |
| **Pyrexia** | - | | - | 6 (18.75) | | 0 (0) |
| **Rash** | 3 (8.8) | | 0 (0) | 3 (9.3) | | 0 (0) |
| **Diarrhea** | 2 (5.9) | | 0 (0) | 2 (6.3) | | 0 (0) |
| **Nausea** | - | | - | 2 (6.3) | | 0 (0) |
| **Fatigue** | 1 (2.9) | | 0 (0) | 2 (6.3) | | 0 (0) |
| **Myalgia** | - | | - | 2 (6.3) | | 0 (0) |
| **Arthalgia** | - | | - | 2 (6.3) | | 0 (0) |
| **Hypothyreoidism** | 1 (2.9) | | 0 (0) | 2 (6.3) | | - |
| **CK increase** | - | | - | 1 (3.2) | | 1 (3.2) |
| **Dermatitis** | - | | - | 1 (3.2) | | 0 (0) |
| **Pneumonitis** | 1 (2.9) | | 1 (2.9) | 1 (3.2) | | 1 (3.2) |
| **Thyreoiditis** | 1 (2.9) | | 0 (0) | - | | - |
| **Cough** | 1 (2.9) | | 0 (0) | - | | - |
| **Arthalgia** | 1 (2.9) | | 0 (0) | - | | - |
| **Colitis** | 1 (2.9) | | 0 (0) | - | | - |
| **Hepatitis** | 1 (2.9) | | 1 (2.9) | - | | - |
| **Amylase+Lipase >ULN** | 1 (2.9) | | 1 (2.9) | - | | - |
| **Meningitis** | 1 (2.9) | | 1 (2.9) | - | | - |
